# Molecular Investigation of Tularemia Outbreaks, Spain, 1997–2008

**DOI:** 10.3201/eid2005.130654

**Published:** 2014-05

**Authors:** Jaime Ariza-Miguel, Anders Johansson, María Isabel Fernández-Natal, Carmen Martínez-Nistal, Antonio Orduña, Elías F. Rodríguez-Ferri, Marta Hernández, David Rodríguez-Lázaro

**Affiliations:** Instituto Tecnológico Agrario de Castilla y León, Valladolid, Spain (J. Ariza-Miguel, M. Hernández, D. Rodríguez-Lázaro);; Umeå University, Umeå, Sweden (A. Johansson);; Complejo Asistencial Universitario de León, León, Spain (M.I. Fernández-Natal);; Laboratorio Regional de Sanidad Animal León, Valladolid (C. Martínez-Nistal);; Universidad de Valladolid, Valladolid (A. Orduña);; Universidad de León, León (E.F. Rodríguez-Ferri);; Universidad de Burgos, Burgos, Spain (D. Rodríguez-Lázaro)

**Keywords:** tularemia, Francisella tularensis subsp. holarctica, bacteria, pulsed-field gel electrophoresis, variable number tandem repeat loci, outbreaks, genotyping, molecular investigation, phylogenetics, zoonoses, Spain

## Abstract

Tularemia outbreaks occurred in northwestern Spain in 1997–1998 and 2007–2008 and affected >1,000 persons. We assessed isolates involved in these outbreaks by using pulsed-field gel electrophoresis with 2 restriction enzymes and multilocus variable number tandem repeat analysis of 16 genomic loci of *Francisella tularensis,* the cause of this disease. Isolates were divided into 3 pulsotypes by pulsed-field gel electrophoresis and 8 allelic profiles by multilocus variable number tandem repeat analysis. Isolates obtained from the second tularemia outbreak had the same genotypes as isolates obtained from the first outbreak. Both outbreaks were caused by genotypes of genetic subclade B.Br:FTNF002–00, which is widely distributed in countries in central and western Europe. Thus, reemergence of tularemia in Spain was not caused by the reintroduction of exotic strains, but probably by persistence of local reservoirs of infection.

## Abstract 2

This disease has reemerged because of persistence of local reservoirs of infection.

Tularemia is a zoonosis caused by the gram-negative bacterium *Francisella tularensis*. *F. tularensis* is a highly contagious facultative intracellular pathogen and has infectious doses as low as 10–50 bacteria; it is transmitted by inhalation, direct contact with infected animals, or ingestion of contaminated water or food. The number of species susceptible to infection by this agent is higher than for any other known zoonotic pathogen (*1*). Because of its potential to cause adverse public health effects and mass casualties by bioterrorist attack, the pathogen is 1 of 6 agents listed as a Tier 1 agent by the US Department of Health and Human Services (*2*).

*F*. *tularensis* includes 4 subspecies (*F*. *tularensis* subsp. *tularensis*, *F*. *tularensis* subsp. *holarctica*, *F*. *tularensis* subsp. *novicida*, and *F*. *tularensis* subsp. *mediasiatica*), which show marked differences in many epidemiologic features, including geographic distribution, virulence, and genetic diversity (*3*). *F. tularensis* subsp. *tularensis* (Jellison type A) and *F. tularensis* subsp. *holarctica* (Jellison type B) are major clinical pathogens. *F. tularensis* subsp. *tularensis* is the most virulent subspecies and can cause life-threatening disease; its distribution seems to be restricted to North America, although a single report indicated its presence in Europe (*4**–**7*). *F*. *tularensis* subsp. *holarctica* causes a less severe disease, and although widespread throughout the Northern Hemisphere, it has restricted genetic diversity, which suggests recent emergence and successful geographic spread (*5**,**7**–**9*).

Tularemia was first reported in Spain in 1997, when it caused one of the largest outbreaks in humans ever described (*10*). Overall, 559 cases were confirmed during June 1997–April 1998 in 10 provinces. The outbreak was associated with hunting and handling of hares (*Lepus europaeus*) in northwestern Spain. The most common clinical form was ulceroglandular tularemia (55.4%); glandular (15.3%) and typhoid forms (6.6%) of the disease also occurred frequently. A second major human outbreak in humans, which affected 507 persons, occurred in the same area in 2007 and 2008, but in a different epidemiologic context. Its timing coincided with a population peak of the common vole (*Microtus arvalis*), and the most frequent clinical forms of the disease were typhoidal and pneumonic (65% of the cases), which is consistent with infection being acquired through inhalation of *F. tularensis* (*11**–**13*). Sporadic tularemia cases and small outbreaks were reported during 2000–2006 in the interval between the 2 major outbreaks in northwestern Spain (*13**,**14*).

We report comparative genetic analyses of *F. tularensis* cultured from humans and animals during the 2 main tularemia outbreaks (1997–1998 and 2007–2008). We also studied *F. tularensis* isolates circulating in Spain during outbreaks with different epidemiologic patterns and investigated whether reemergence of the pathogen after 10 years of no epidemiologic activity was caused by introduction of exotic strains or by establishment of the pathogen in local reservoirs of infection.

## Methods

### *F. tularensis* Isolates, Culture Conditions, and Biochemical Characterization

We studied 109 *F*. *tularensis* isolates: 37 animal and human *F. tularensis* subsp. *holarctica* isolates from the first outbreak in northwestern Spain (1997–1998); 61 animal and human isolates from the second tularemia epidemic in the same area (2007–2008); 10 10 isolates obtained in the Czech Republic; and reference strain *F*. *tularensis* subsp. *tularensis* Schu (CAPM 5600). Source of isolates, subspecies, host, geographic origin, and year of isolation are shown in the Technical Appendix.

All isolates were grown on modified Thayer-Martin agar plates containing 36 g/L GC agar base, 10 g/L soluble hemoglobin powder, and 2 vials/L Vitox supplement (Oxoid, Basingstoke, UK) at 37°C for 2–3 days in aerobic conditions. Biochemical characterization included tests for oxidase and catalase activities, glucose and glycerol fermentation, and urea hydrolysis.

### Genetic Characterization

Species and subspecies were identified by real-time and conventional PCRs specific for the *fopA* gene and the region of difference 1 (RD1) as described (*15**,**16*). RD23 was analyzed by PCR for identification of a genetic group of isolates that had been found on the Iberian Peninsula (*17*).

Pulsed-field gel electrophoresis (PFGE) and multilocus variable number tandem repeat analysis (MLVA) were used to classify isolates into genetic subpopulations. The PFGE protocol described (*18*), which used restriction enzymes *Xho*I and *Bam*HI, was optimized to provide major improvements in quality of fingerprint patterns.

Bacterial cells were suspended in SE buffer (25 mmol/L EDTA, 75 mmol/L NaCl, pH 7.5) to an absorbance of 0.5–0.6 at 600 nm. Cells were lysed in agarose plugs and plugs were washed 5 times with Tris-EDTA buffer (10 mmol/L Tris-HCl, 1 mmol/L EDTA, pH 8.0) for 30 min at 50°C. DNA in the plugs was digested with 40 U of *Xho*I (New England Biolabs, Ipswich, MA, USA) or 40 U of *Bam*HI (New England Biolabs), for 16 and 3 h, respectively, at 37°C following the manufacturer’s protocol. DNA fragment sizes were determined by electrophoresis and by comparing bands with a Lambda Ladder PFG Marker (New England Biolabs).

MLVA was performed as described for 16 variable number tandem repeat loci (*5*). To ensure analysis of identical genetic material by PFGE and MLVA, we used DNA from the same culture for both methods. MLVA markers were amplified by using PCR, and sizes of amplification products were determined by electrophoresis on 3.5% high-resolution agarose MS-8 gels (Conda Pronadisa, Madrid, Spain), except for Ft-M3, Ft-M21, Ft-M22, and Ft-M24 MLVA markers, for which the sizes were determined by using capillary electrophoresis. At least 2 alleles were sequenced for each MLVA marker to confirm that size differences observed resulted from the expected variations in numbers of tandem repeats. Forward and reverse sequences were aligned by using MEGA v.4 software (*19*), and consensus sequences were used to predict the number of tandem repeats in each allele.

### Data Analyses

Simpson index of diversity, which measures the probability that 2 unrelated strains from the test population will be classified into different typing groups (*20*), was calculated to compare the discriminative power of PFGE typing with that of MLVA for assessing genetic diversity among isolates. The adjusted Wallace coefficient for quantification of agreement between PFGE typing and MLVA results was also calculated. Both analyses were performed by using Comparing Partitions (*21*).

PFGE patterns were analyzed by using Bionumerics 6.6 (Applied-Maths NV, Sint-Martens-Latem, Belgium) to describe genetic relationships among isolates. Dendrograms were constructed by using the Dice similarity coefficient and the unweighted pair group mathematical average clustering algorithm. MLVA data, expressed as allelic profiles for isolates, were analyzed by using Bionumerics 6.6. Minimum spanning trees were calculated with priority rules set at first link allelic profiles and maximum numbers of single-locus variants and then maximal numbers of single-locus variants and double-locus variants. MLVA types were classified as members of a clonal complex if they had the same allele at15 of the 16 MLVA markers. A map of the distribution of isolates showing the geographic origin and number of isolates per province was generated by using Arcgis 9.2 software (ESRI, Redlands, CA, USA).

## Results

### Subspecies and Genetic Subclade of *F. tularensis* Isolates

All isolates were negative for oxidase activity, weakly positive for catalase activity, and positive for acid production from glucose; none of the isolates hydrolyzed urea. Only the reference strain *F. tularensis* subsp. *tularensis* Schu (CAPM 5600) produced acid from glycerol. Real-time PCR specific for the *fop*A gene and size determination at the RD1 region showed that all isolates from Spain and the Czech Republic were *F. tularensis* subsp. *holarctica* (Technical Appendix). All isolates from Spain included in this study had the 1.59-kbp deletion at the RD23 loci, which is characteristic of the *F. tularensis* subsp. *holarctica* genetic subclade B.Br:FTNF002–00 (also known as the Iberian clone or the central and western European genetic group).

### Characterization by PFGE

All 107 *F*. *tularensis* subsp. *holarctica* isolates showed the same fingerprint pattern by PFGE with the restriction enzyme *Xho*I, irrespective of their geographic origin, host, or date of isolation (isolate TU41 was not typeable by PFGE analysis). This pattern consisted of ≈20 DNA fragments >70 kbp. The *F*. *tularensis* subsp. *tularensis* strain Schu (CAPM 5600) showed a different banding pattern.

**Figure 1:**
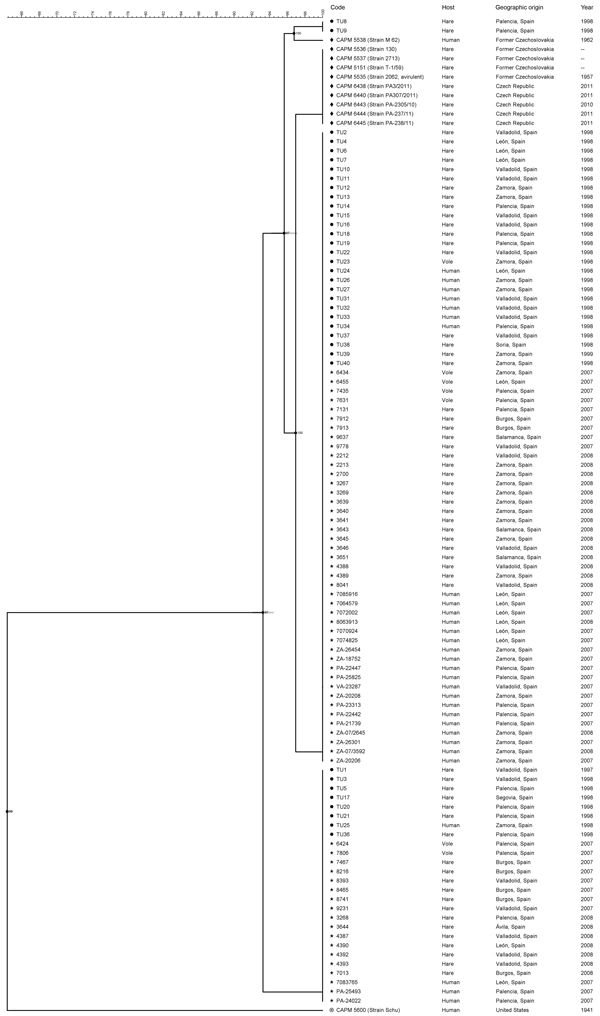
Genetic relationships among 108 *Francisella tularensis* isolates based on comparison of pulsed-field gel electrophoresis (PFGE) profiles obtained with restriction enzyme *Bam*HI. The dendrogram was produced by using a Dice similarity coefficient matrix with unweighted pair group method with arithmetic mean. All isolates, exept TU30, are *F. tularensis* subsp. *holarctica*. TU30 is *F. tularensis* subsp. tularensis. Scale bar indicates similarity values.

In contrast, PFGE with the restriction enzyme *Bam*HI discriminated 5 genotypes among the *F*. *tularensis* subsp. *holarctica* isolates. *F*. *tularensis* subsp. *tularensis* strain Schu (CAPM 5600) showed a highly unrelated banding pattern with maximal pairwise distance to all other isolates. The *Bam*HI patterns consisted of 20–24 DNA fragments with a size range of 20–245 kbp. All *F*. *tularensis* subsp. *holarctica* genotypes were closely related (93.3% similarity), and there were only 1-band differences between pulsotypes (Figure 1).

Sixty-nine (63.9%) isolates from Spain clustered into pulsotype A and 26 (24.1%) other isolates from Spain clustered into pulsotype B. All isolates from the Czech Republic, except for isolate CAPM 5538, had the same fingerprint pattern, which was designated pulsotype D (8.3%). Isolate CAPM 5538 showed a pulsotype that clustered with the 2 remaining isolates from Spain (TU8 and TU9) in pulsotype C (1.9%). One isolate from Spain, TU41, could not be genotyped by PFGE despite several attempts (Technical Appendix).

There were some discrepancies between our findings and those reported for the 37 isolates in Spain from 1997 (*18*). Improvements in quality of fingerprint patterns enabled us to distinguish between isolates from Spain and those from the Czech Republic by using PFGE and restriction enzyme *Bam*HI. Furthermore, isolates TU3, TU17, TU21, and TU25 were unequivocally assigned to pulsotype B instead of pulsotype A. In instances of discrepancy, analyses were repeated in triplicate with new cultures, and the findings reported were confirmed. Distribution of the 107 *F*. *tularensis* subsp. *holarctica* isolates into 4 pulsotypes resulted in a Simpson index of diversity of 0.522. There was no obvious correlation between the pulsotype of an isolate from Spain and its geographic origin, host, or tularemia outbreak with which it was associated.

### Characterization by MLVA

**Figure 2:**
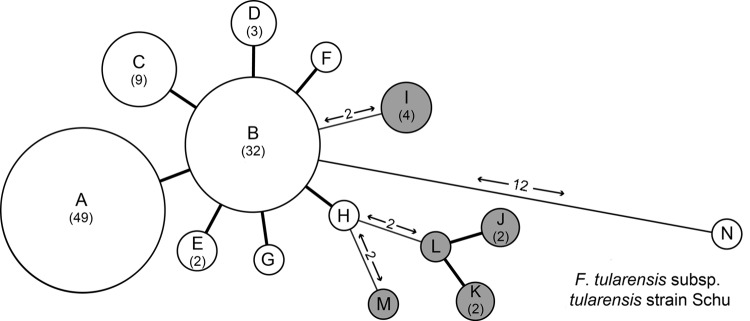
Minimum-spanning tree based on multilocus variable number of tandem repeat analysis (MLVA) genotypes, showing genetic relationships among 98 *Francisella tularensis* subsp. *holarctica* isolates from Spain (white circles), 10 *F. tularensis* subsp. *holarctica* reference isolates from the Czech Republic (gray circles), and reference strain *F. tularensis* subsp. *tularensis* Schu (CAPM 5600). Each node represents a unique MLVA type, and size is proportional to the number of isolates with that genotype (values in parentheses). Numbers on lines between nodes indicate number of typing markers that were different between genotypes. A 1-marker difference is indicated by a thick line.

The allele-based analysis of genetic relationships identified 13 MLVA types among the 108 *F*. *tularensis* subsp. *holarctica* isolates and showed that *F*. *tularensis* subsp. *tularensis* strain Schu (CAPM 5600) was more distantly related (Figure 2). The 10 isolates from the Czech Republic were assigned to 5 MLVA types, which differed from isolates from Spain by ≥2 alleles. Marker Ft-M3 provided the highest number of alleles (*6*). Six copy numbers were detected among the 109 isolates; for Ft-M6, Ft-M9, and Ft-M20, there were 3 copy numbers. Markers Ft-M5, Ft-M7, Ft-M8, Ft-M10, Ft-M13, FT-M16, Ft-M19, Ft-M21, Ft-M22, Ft-M23, and Ft-M24 each had 2 alleles: these markers with 2 alleles, except for Ft-M24, discriminated only *F*. *tularensis* subsp. *tularensis* strain Schu (CAPM 5600) from all isolates of *F*. *tularensis* subsp. *holarctica* (Table).

**Table:** Multilocus variable number tandem repeat analysis of 98 *Francisella tularensis* isolates from Spain and 11 reference isolates*

MLVA genotype	No. isolates (origin)	No. MLVA markers that discriminated *F. tularensis* subsp. *holarctica* isolates				
		Ft-M3	Ft-M6	Ft-M9	Ft-M20	Ft-M24†
A	49 (Spain)	5	4	2	3	2 (∆16 bp)
B	32 (Spain)	4	4	2	3	2 (∆16 bp)
C	9 (Spain)	6	4	2	3	2 (∆16 bp)
D	3 (Spain)	3	4	2	3	2 (∆16 bp)
E	2 (Spain)	4	7	2	3	2 (∆16 bp)
F	1 (Spain)	4	4	2	4	2 (∆16 bp)
G	1 (Spain)	4	4	3	3	2 (∆16 bp)
H	1 (Spain)	7	4	2	3	2 (∆16 bp)
I	4 (former Czechoslovakia)	4	6	2	3	2
J	2 (Czech Republic)	5	7	2	3	2
K	2 (Czech Republic)	6	7	2	3	2
L	1 (former Czechoslovakia)	7	4	2	2	2
M	1 (Czech Republic)	7	7	2	3	2
N‡	1 (United States)	28	4	4	3	1

*MLVA, multilocus variable number tandem repeat. †All isolates from Spain had the unique 16-bp deletion at marker FT-M24 that is characteristic of genetic subclade B.Br:FTNF002–00 (Iberian clone). ‡Genotype N, *F. tularensis* subsp. *tularensis* strain Schu, showed additional unique alleles at the following MLVA markers: Ft-M5, Ft-M7, Ft-M8, Ft-M10, Ft-M13, Ft-M16, Ft-M19, Ft-M21, Ft-M22, and Ft-M23.

Ft-M24 had a 464-bp allele that was found in all isolates from Spain analyzed in this study, but was not present in any other isolates. Ft-M24 has been found only in isolates of genetic subclade B.Br:FTNF002–00 (the Iberian clone or the central and western European genetic group). Sequence analysis of the Ft-M24 DNA fragment showed that the unique allele size was caused by deletion of a 16-bp sequence adjacent to 2 copies of the Ft-M24 tandem repeat (GenBank accession no. KC696513). For marker Ft-M12, because all 109 isolates had the same copy number, this marker provided no typing resolution. Distribution of *F*. *tularensis* subsp. *holarctica* isolates among 12 MLVA types was uneven; >70% of the isolates in the MLVA types A (49 isolates, 45%) and B (32 isolates, 29.4%) (Table). Simpson index of diversity, which showed the discriminatory power of MLVA for the 108 *F*. *tularensis* subsp. *holarctica* isolates, was 0.708.

**Figure 3:**
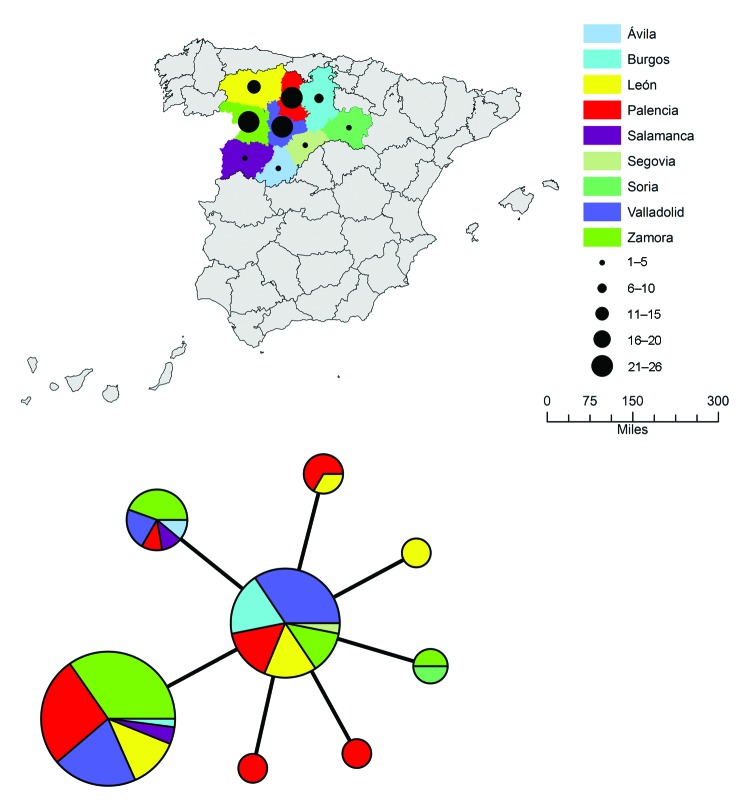
A) Geographic distribution of 98 *Francisella tularensis* subsp. *holarctica* isolates from Spain. Color codes represent geographic origin, and black circles represent number of isolates recovered per province. B) Minimum-spanning tree based on multilocus variable number tandem repeat (MLVA) analysis of genotypes, showing genetic relationships among 98 *F*. *tularensis* subsp. *holarctica* isolates from Spain. Each circle represents a unique MLVA type and size of each node is proportional to the number of isolates of that type. The MLVA types are colored according to the geographic origins of the isolates with the same color code as in panel A.

The 98 isolates from Spain were classified into 8 MLVA types, which essentially grouped as 2 closely related clonal complexes that differed at only 1 of the 16 MLVA markers (Figure 2). All MLVA types from Spain were single-locus variants of MLVA type B, which indicated this type was the founder genotype of *F. tularensis* that caused tularemia in northwestern Spain. No clear relationship was found between genotype and geographic origin (Figure 3), source of infection, or host in Spain. The same genotype was usually isolated from hares, voles, and humans in Spain.

**Figure 4:**
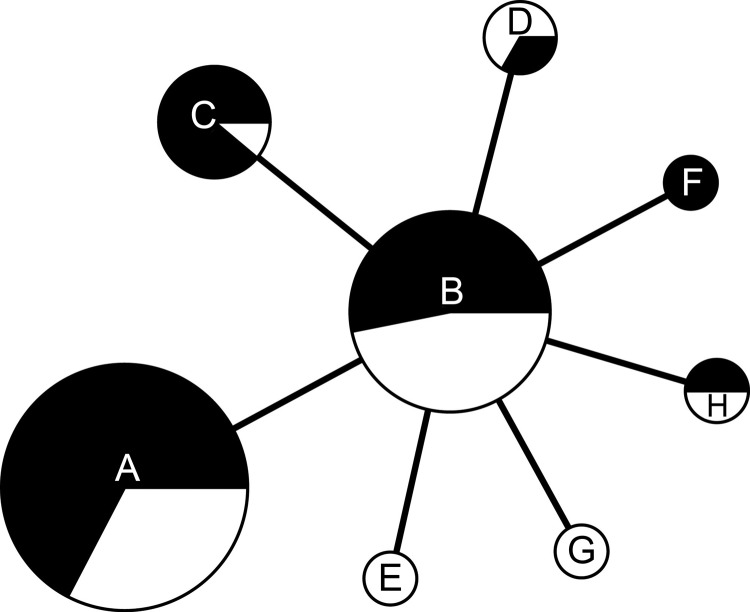
Minimum-spanning tree based on multilocus variable number tandem repeat (MLVA) analysis of genotypes showing genetic relationships among 98 *Francisella tularensis* subsp. *holarctica* isolates from Spain with reference to 2 human tularemia outbreaks in 1997–1998 and 2007–2008, respectively. White sections in circles indicate *F*. *tularensis* subsp. *holarctica* isolates recovered during the first human tularemia outbreak (1997–1998), and black sections indicate isolates recovered during the second outbreak (2007–2008). Each circle represents a unique MLVA type and size is proportional to the number of isolates of that type.

Comparison of isolates from the 2 outbreak periods (37 isolates for 1997–1998 and 61 isolates for 2007–2008) showed that the same *F. tularensis* genotypes caused tularemia in both outbreaks (Figure 4). Isolates from the second outbreak showed less genetic diversity than those from the first outbreak (Simpson indices 0.62, 95% CI 0.53–0.71 and 0.66, 95% CI 0.57–0.75, respectively; the difference was not significant at the 95% level). Comparison of allele distribution at the most variable marker (Ft-M3) showed an overall similarity between isolates causing the outbreaks, although the most common copy number was 4 during the first outbreak and 5 during the second outbreak, which might indicate a stepwise increase in copy number over time. Overall, our findings for 37 isolates from the first outbreak were consistent with the data reported by Dempsey et al. (*17*) although there were 2 discrepancies. First, isolate TU18 had a unique allele with 3 tandem repeats at Ft-M9, which distinguished this isolate from all other *F*. *tularensis* subsp. *holarctica* isolates. Second, isolate TU31 had the same FT-M10 allele as all other isolates. We confirmed our results for these discrepancies in triplicate.

### Quantification of Agreement between PFGE Typing and MLVA

Congruence of the 2 methods (PFGE typing and MLVA) for isolate classification was weak for the 107 *F. tularensis* subsp. *holarctica* isolates (1 of the 108 isolates was excluded because it was not typeable by PFGE analysis). This finding was true for reverse comparisons of both methods: if 2 isolates were in the same PFGE pulsotype; they had an 18% chance of having the same MLVA type. Conversely, having the same MLVA type was associated with a 40% chance of having the same PFGE pulsotype. The adjusted Wallace coefficient for PFGE versus MLVA was 0.18 (95% CI 0.04–0.32) and that for MLVA versus PFGE was 0.40 (95% CI 0.20–0.58).

## Discussion

*F*. *tularensis* subsp. *holarctica* has shown limited genetic diversity worldwide (*5**,**7**,**22**,**23*). This finding might be the result of a relatively recent bottleneck or clonal expansion event that drastically reduced genetic variation of the bacterial population (*5**,**7*). Consistent with previous findings, we observed extremely limited genomic diversity among the 98 *F*. *tularensis* subsp. *holarctica* isolates from Spain analyzed by 2 genotyping tools: PFGE with 2 restriction enzymes and MLVA at 16 highly variable tandem repeat loci. PFGE identified 3 genotypes with single band differences (93.3% similarity) (Figure 1). MLVA discriminated these isolates into 8 MLVA types, but in pairwise comparisons they differed at no more than 2 of 16 MLVA markers. Thus, our collection shows extreme genetic homogeneity (Figure 2).

An *F. tularensis* subsp. *holarctica* lineage in central and western Europe (France, Spain, and Switzerland) has been defined (*7**,**17**,**24**,**25*). Strains belonging to this lineage have 2 unique genetic traits: a 1.59-kbp genomic deletion at the RD23 locus and a unique 464-bp allele at Ft-M24. All isolates from Spain had these alleles, irrespective of the outbreak, geographic origin, or the host from which they were recovered. Thus, all isolates from Spain analyzed belong to the genetic subclade B.Br:FTNF002–00 (the Iberian clone or central and western European genetic group) (*7*). Furthermore, all MLVA types for isolates from Spain were single-locus variants of MLVA type B, which suggested that this type might be a founding genotype that has evolved into multiple other genotypes that differ from the founding genotype at a single loci. In this scenario, all strains causing tularemia outbreaks in Spain are linked to this founder (ancestral) genotype.

We found poor congruence between typing results of PFGE and MLVA for 107 *F. tularensis* subsp. *holarctica* isolates. However, the 2 methods might indicate different types of genetic variation. PFGE is a suitable approach for detecting rearrangements in a genome, and differences of only 1 band observed among the 98 isolates from Spain are presumably consequences of a single mutation event that might be an inversion, translocation, deletion, or a single-nucleotide polymorphism (*26*). In contrast, MLVA detects variation in several, fast-evolving, repeated sequences. However, such rapidly evolving sequences are susceptible to homoplasy, and genetic classification of isolates on the basis of a difference at only 1 of 16 genetic loci, as for isolates from Spain, could be biased because of genetic reversion events.

Because some of the mutations detected by PFGE or MLVA might not be selectively neutral, we might have observed time-dependent mutations that are transient on evolutionary time scales and will frequently be eliminated by selection pressure acting on them (*27*). If this hypothesis is true, use of single mutations for genetic discrimination may lead to incorrect phylogenetic inferences. Therefore, poor congruence between PFGE and MLVA for identifying genetic subclade B.Br:FTNF002–00 of *F*. *tularensis* subsp. *holarctica* in Spain might be caused by limited bacterial diversity. Use of more extensive genetic analyses for typing, such as whole genome sequencing, might be useful in subsequent molecular epidemiology studies.

In Spain, tularemia was first reported in late 1997 in association with one of the largest human outbreaks ever described (*10*). The most common route of infection of humans was by direct contact when hunting and handling hares (*L. europaeus*). Consistent with infection with *F. tularensis* through the skin, the most frequent clinical form was ulceroglandular tularemia (55.4%); glandular (15.3%) and typhoid (6.6%) forms of the disease were also observed. A second major human outbreak occurred in the same geographic area in northwest Spain in 2007 and 2008 after 10 years of no epidemiologic activity. The epidemiology of the second outbreak was different from that of the first outbreak. The second outbreak occurred when the population of the common vole (*Microtus arvalis*) peaked, and >65% of case-patients had typhoidal and pneumonic forms of tularemia (*12**,**13*), which is consistent with infection by inhalation.

Few outbreaks of tularemia caused by airborne transmission of the bacteria have been reported. These outbreaks include a notable outbreak of inhalational tularemia in Sweden in 1966–1967 associated with environmental exposure of farmers in which >600 cases were diagnosed (*28*).There were clusters of outbreaks on Martha’s Vineyard (Massachusetts, USA) in 1978 and 2000 (*29**,**30*) and cases of tularemia caused by airborne transmission to 53 farmers in northern Finland during 1982. (*31*). In Germany in 2005, a total of 39 participants in a hare hunt were infected after exposure to contaminated droplets generated by rinsing infected hares (*32*).

Isolates from Spain obtained during the second tularemia outbreak had the same genotypes as those obtained during the first outbreak (Figures 1, 4). Furthermore, we did not observe any relationships between genotype and geographic origin, or host from which the isolates were recovered, which suggested that in that area, the same clones were circulating in all hosts (Figure 3, panel B). These results are useful because the 2 outbreaks had substantial epidemiologic differences (*10**,**12*). Our findings indicate that the outbreak in 2007, after 10 years of no epidemiologic activity, was not caused by introduction of a new strain, but by reemergence of an endemic bacterial population that has been circulating in the region for at least the past 15 years. Furthermore, our findings also suggest that clinical forms of the outbreak are determined by ecologic processes involved in infection (e.g., route of infection, infective dose) rather than by the genotype of the pathogen.

In conclusion, we report genetic characterization of *F*. *tularensis* subsp. *holarctica* isolated in Spain during 2 of the largest tularemia outbreaks worldwide. There were marked epidemiologic differences between the 2 outbreaks, which were separated by 10 years of no epidemic activity. Molecular investigations showed that both outbreaks were caused by the same group of closely related genotypes in subclade B.Br:FTNF002–00. Therefore, the reemergence of tularemia in 2007 was presumably not caused by introduction of a new strain, but by persistence of local reservoirs of infection. These findings, along with sporadic cases of tularemia in 1998 and 2007, suggest that local foci of tularemia have become established in Spain. Further investigations will help identify these endemic foci and clarify biotic and abiotic factors that have favored establishment of the pathogen in northwestern Spain.

Technical Appendix Information on *Francisella tularensis* subsp. *holarctica* isolates used in this study and results of PFGE and MLVA.
